# Detection of Oenological Polyphenols via QCM-D Measurements

**DOI:** 10.3390/nano12010166

**Published:** 2022-01-04

**Authors:** Mariacristina Gagliardi, Giorgia Tori, Matteo Agostini, Francesco Lunardelli, Fabio Mencarelli, Chiara Sanmartin, Marco Cecchini

**Affiliations:** 1 NEST, Istituto Nanoscienze-CNR and Scuola Normale Superiore, Piazza San Silvestro 12, I-56127 Pisa, Italy; mariacristina.gagliardi@nano.cnr.it (M.G.); g.tori2@studenti.unipi.it (G.T.); francesco.lunardelli@sns.it (F.L.); 2 INTA S.r.l., Intelligent Acoustics Systems, Via Nino Pisano 14, I-56122 Pisa, Italy; m.agostini@intasystems.net; 3 UNIPI Department of Agriculture Food Environment, University of Pisa, Via Del Borghetto 80, I-56124 Pisa, Italy; fabio.mencarelli@unipi.it (F.M.); chiara.sanmartin@unipi.it (C.S.); 4 Interdepartmental Research Center, Nutraceuticals and Food for Health, University of Pisa, Via Del Borghetto 80, I-56124 Pisa, Italy

**Keywords:** polyphenols, quartz crystal microbalance, biosensor, functionalization, precision oenology, acoustic wave sensor

## Abstract

Polyphenols are a family of compounds present in grapes, musts, and wines. Their dosage is associated with the grape ripening, correct must fermentation, and final wine properties. Owing to their anti-inflammatory properties, they are also relevant for health applications. To date, such compounds are detected mainly via standard chemical analysis, which is costly for constant monitoring and requires a specialized laboratory. Cheap and portable sensors would be desirable to reduce costs and speed up measurements. This paper illustrates the development of strategies for sensor surface chemical functionalization for polyphenol detection. We perform measurements by using a commercial quartz crystal microbalance with dissipation monitoring apparatus. Chemical functionalizations are based on proteins (bovine serum albumin and gelatin type A) or customized peptides derived from istatine-5 and murine salivary protein-5. Commercial oenological additives containing pure gallic tannins or proanthocyanidins, dissolved in water or commercial wine, are used for the analysis. Results indicate that selected functionalizations enable the detection of the two different tannin families, suggesting a relationship between the recorded signal and concentration. Gelatin A also demonstrates the ability to discriminate gallic tannins from proanthocyanidins. Outcomes are promising and pave the way for the exploitation of such devices for precision oenology.

## 1. Introduction

Gravimetric sensors based on acoustic waves are fast and reliable tools for a high-precision detection of mass, viscosity, conductivity, and density [1]. Sauerbrey reported, for the first time in 1959, the detection of a substance deposited on a vibrating element (resonator) via acoustic devices [2]. To date, acoustic transducers find application in the manufacturing of a variety of sensors, such as the quartz crystal microbalance (QCM), Rayleigh wave sensors, shear-horizontal surface acoustic wave sensors, Love wave sensors, and several other devices. The QCM is a tool using a piezoelectric resonator to generate bulk acoustic waves (BAW). BAWs propagate inside the resonator and their frequency changes when an event, such as the adsorption of a molecule over sensor surface or a chemical reaction involving the analyte, occurs [3,4]. BAWs in QCM have a shear-horizontal polarization, and this makes them compatible with real-time measurements in a liquid environment [5]. In a typical biosensor, a biologically active molecule (probe) decorates a surface exposed to the sample [6,7], and interacts with the analyte [8]. Due to their versatility and high performance, acoustic-wave-based biosensors find a variety of applications, such as for the detection of proteins [9,10,11] and enzymes [12,13,14] and for fast diagnostics [15]. To date, there has been a growing interest in the exploitation of acoustic sensors in food science [16], opening the way to new sciences such as precision oenology [17].

Polyphenols are a family of compounds present in grapes, musts, and wines. Their dosage is associated with the grape ripening, correct must fermentation, and final wine properties. Owing to their anti-inflammatory properties, they are also relevant for health and have the potential to reduce risks for cardiovascular, or other non-communicable diseases [18,19,20]. Wine is a complex matrix containing several hundreds of compounds such as, among others, water, ethanol, organic acids, carbohydrates, and polyphenols [21,22]. Tannins and anthocyanins are extremely important polyphenols, especially for red wines [23], determining most of their organoleptic properties [24]. Currently, polyphenol concentration is monitored by sampling berries, must, or wines [25,26]. Collected samples are afterwards analyzed by a traditional chemical analysis [27,28]. The extensive monitoring of winemaking process is ideally needed to obtain high quality products, but associated costs would be too high. Easy-to-use and low-cost analytic tools can overcome this limitation.

QCM-D was already used for wine analysis, with ad hoc experiments exploring the role of positively and negatively charged functional groups [29] and different polymeric functionalizations [30]. Cited works have demonstrated that hydrophilic and negatively charged groups (–SO_3_H and –COOH) have the best ability to adsorb red wine constituents, while the hydrophilic non charged groups –OH show the worst performance. On the other hand, in polymeric layers, –NH_2_ and –COOH promoted adsorption of constituents from white wines, –OH exhibited a strong preference for rosé wines and the acrylic acid for the red wine. These results indicate that the selective adsorption of components over the functional layer is a complex phenomenon that does not depend only on the exposed functional groups. QCM-D was also used for the analysis of potential wine contaminations [31], and to study the interactions between wine compounds and salivary proteins [32,33,34], with the fascinating aim to compare measured data with the human mouthfeel [35]. To the best of our knowledge, QCM-D has not yet been used for wine polyphenol detection.

A valid probe molecule for wine polyphenol detection can be selected among the agents used for wine refinement, a process performed to make the final product clear [36] and less subject to oxidation [37]. Tannic acid, the unit constituting several polyphenols, forms colloidal solutions when it interacts with molecules forming hydrogen bonds [38]. Proteins can establish Van der Waals interactions [39], thus are used in wine refinement for their ability to form complexes with polyphenols, giving stable colloidal solutions [40].

In this work, we present a potentially powerful, cheap, and fast approach for the quantification of polyphenols. With this aim, we investigate four functionalizations for QCM-D sensors: (1) bovine serum albumin (BSA); (2) type A gelatin for porcine skin (Gel-A); (3) the synthetic low-molecular-weight peptide called istatine-5 (Ist-5); and (4) a peptide fragment of the murine salivary protein-5 (MP-5). Such functionalizations are tested with watery and winery solutions, containing polyphenols. BSA, already tested in QCM-D experiments with gallic tannins from green tea [41,42], can interact with free polyphenols forming soluble complexes [43] with a high efficiency [44], and the complexation mechanism is already well known [45]. Moreover, BSA has a great ability to form complexes with high-molecular-weight polyphenols [46], and this ability makes BSA also a good candidate in the detection of tannins condensed after oxidative processes. Gel-A is used to lower the overall polyphenols content in wines, reducing astringency more than other proteins [47,48]. This protein has already been used in QCM-D experiments to evaluate the astringency in beer [49]. Ist-5 is a small peptide with a larger ability to precipitate tannins than proline-rich proteins [50]. Its aminoacidic sequence has a potential to form useful interactions with tannins [51], establishing strong interactions with the aromatic rings of condensed proanthocyanidin tannins [52]. MP-5 is a proline-rich peptide derived from the whole murine salivary protein-5 [53], and it has great affinity for hydrolysable gallic tannins [54].

## 2. Materials and Methods

### 2.1. Reagents

1,4-Dithiothreitol (DTT) was used as reducing agent for thiolated molecules (Sigma Aldrich, St. Louis, MO, USA); 12-mercaptododecanoic acid (12-MCA, M_w_ 232.4 Da, purity degree 96%) as linker for proteins (Sigma Aldrich); bovine serum albumin (BSA, M_w_ 66.5 kDa) and gelatin from porcine skin type A (Gel-A, High bloom M_w_ 75.0 kDa) as functionalizing proteins (Sigma Aldrich); istatine-5 (Ist-5, amino acid sequence: DSHAKRHHGYKRKFHEKHHSHRGYC, M_w_ 3139 Da) and murine salivary protein-5 (MP-5, GPQQRPPQPGNQQGPPPQGGPQC, M_w_ 2350 Da) with C-terminal cysteine (both synthesized by Biomatik Kitchener, Ontario, Canada, purity degree 95%) as functionalizing peptides; N-(3-Dimethylaminopropyl)-N′-ethylcarbodiimide hydrochloride (EDCl) and N-hydroxysuccinimide (NHS) as coupling agents (Sigma Aldrich, purity degree ≥ 98%); commercial Italian wine Tavernello (production lot no. LA1050MO), oenological tannin commercial blends TANIN GALALCOOL (TG, polyphenol content > 95%), TANIN VR SUPRA (TS, >65%) TANIN GALALCOOL SP (TGSP, >95%), TANIN VR GRAPE (TGR, >65%) (LAFFORT^®^, Bordeaux, France), and Enartis TAN BLANC (TB, Esseco S.r.l., San Martino Trecate NO, Italy, Enartis Division, polyphenol content n.a.) for the preparation of samples. All reagents were used as received.

### 2.2. Quartz Crystal Microbalance with Dissipation Monitoring (QCM-D) Experiments

QCM-D (E4 model, Q-Sense AB, Sweden) measurements were performed with polished AT-cut quartz crystals (gold electrodes, fundamental resonance frequency *f*_0_ = 5 MHz, diameter = 14 mm, thickness = 100 nm) in static mode (stop flow), with fluidic cells thermostated at 25 °C. This apparatus allows recording simultaneously the resonance frequency shift (Δ*f*) and energy dissipation (Δ*D*) for up to 13 overtones by exciting the fundamental resonance frequency of the crystal. In this work, the 3rd overtone was chosen as the most sensitive and stable among the entire dataset. In addition, we checked if Δ*D* values were suitable for the application of the Sauerbrey model. According to [55], one of the proposed criteria to check is to have Δ*D* < 2.0 × 10^−6^. Δ*D* values are strictly related to the mechanical behavior of the functionalization adlayer. In our case, the Sauerbrey equation would only be valid for the functionalization with peptides. However, our main interest is in the sample detection; thus, we decided to use the Sauerbrey model also for proteins, with a slight underestimation of calculated areal masses.

### 2.3. Sensor Surface Functionalization

The gold surface of the crystal quartz was modified by covalently attaching the functional layer on the surface. The covalent bonds were obtained by the thiol-gold chemistry.

**Figure 1:**
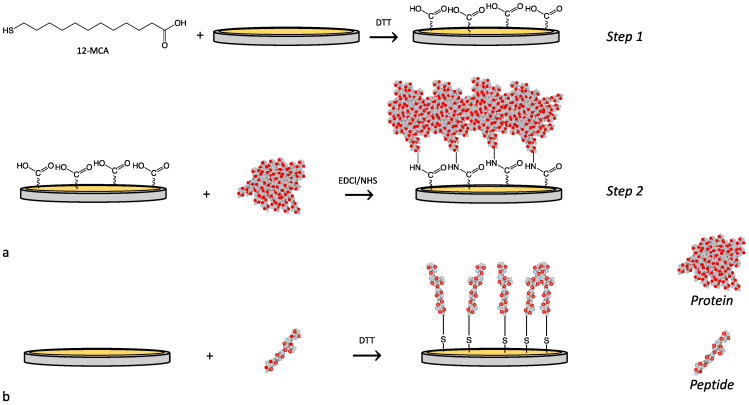
QCM-D functionalization strategies: (**a**) two-steps functionalization with proteins, (**b**) functionalization with peptides.

For the functionalization with proteins that do not contain free thiol groups for the thiol-gold reaction, the 12-MCA was used as linker in a two-step functionalization (Figure 1a). 12-MCA was solubilized (1 mg mL^−1^) in a 1:1 *v*/*v* water/ethanol mixture containing DTT (3.5x mol/mol in respect to free -SH groups). This solution was injected in the QCM-D chamber and data were acquired for 60 min in static mode, then the sensors were rinsed first injecting a 1:1 water/ethanol solution (data acquisition 5 min), then pure water (5 min). This procedure allowed obtaining a 12-MCA self-assembled monolayer (SAM) with the carboxylic functionalities exposed toward the water phase, and available for the following protein conjugation. In the second functionalization step, BSA or Gel-A were dissolved (1 mg mL^−1^) in a solution of EDCl/NHS (50 mM) in ultrapure water. Solutions were injected in the QCM-D chamber and data were acquired for 60 min, then the sensors were rinsed with ultrapure water (10 min). For sensor functionalization with peptides (Figure 1b), Ist-5 or MP-5 solutions in water (1 mg mL^−1^) containing DTT (3.5x mol/mol in respect to free -SH) were injected in the QCM chamber (60 min) then rinsed with water (5 min). Prior to use, quartz crystals were treated with plasma oxygen (Femto Diener) for 10 min at a power of 100 W, immersed in a 5:1:1 solution of water, ammonia (32% *v*/*v*), and oxygen peroxide (25% *v*/*v*) at 75 °C for 15 min, rinsed with water and after with isopropanol, and treated with plasma oxygen again (10 min, 100 W).

### 2.4. Detection Experiments

Aqueous solutions of TG and TS were obtained dissolving the tannin blends in water. Solutions with fixed tannin concentrations (Table 1) were prepared, filtered with a 1.2 μm filter syringe, and the pH was measured. Eventually, samples were stored at 4 °C before use.

Sample analysis of watery samples was performed by injecting three TG or TS solutions at increasing concentrations. Data were acquired in static flow for 15 min for each injected sample, then the sensors were rinsed with water and data acquired for a further 5 min.

Winery samples (Table 2) were prepared by adding the tannin blends in a commercial white wine, previously characterized following the official methods (OIV 2021) (alcoholic degree 10.41 ± 0.01% vol.; pH = 3.16 ± 0.01; titratable acidity = 5.30 ± 0.02 g/L as tartaric acid; net volatile acidity = 0.21 ± 0.01 g/L as acetic acid). Samples were then stabilized, filtered with a 1.2 μm filter syringe, the pH was measured, then samples were stored at 4 °C before use.

Polyphenol concentrations in samples were measured via Folin–Ciocalteu and UV–vis spectroscopy (absorbance at 280 nm), while proanthocyanidins were quantified with the Bate–Smith method [55,56,57].

Measurements of winery solutions were performed by injecting one single sample. Data were acquired in static flow for 15 min, then the sensors were rinsed with water and data acquired for a further 5 min.

### 2.5. Data Analysis

At least two experiments were performed for each condition. Δ*f* and Δ*D* were continuously recorded throughout the whole experiment (sensor, functionalization, detection, and related rinsing). Data are reported as Δ*f* (Hz), Δ*D*, and molar areal mass (pmol cm^−2^). Reported values are the mean values of replicates. Range error bars encompass the lowest and highest values recorded for each condition. Inner fences for large datasets from functionalization experiments were calculated by multiplying the interquartile range (Q1–Q3) by 1.5.

## 3. Results

### 3.1. Functionalization

**Figure 2:**
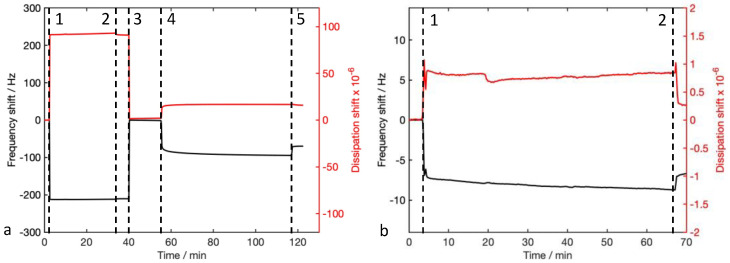
QCM traces of functionalization with (**a**) proteins, (**b**) peptides; in functionalization with proteins, 1: injection of the 12-MCA solution, 2: rinsing with EtOH/water, 3: rinsing with water, 4: injection of the protein solution (in this example, BSA), 5: rinsing with water; in functionalization with peptides, 1: injection of the peptide solution (in this example, MP-5), 2: rinsing with water.

**Figure 3:**
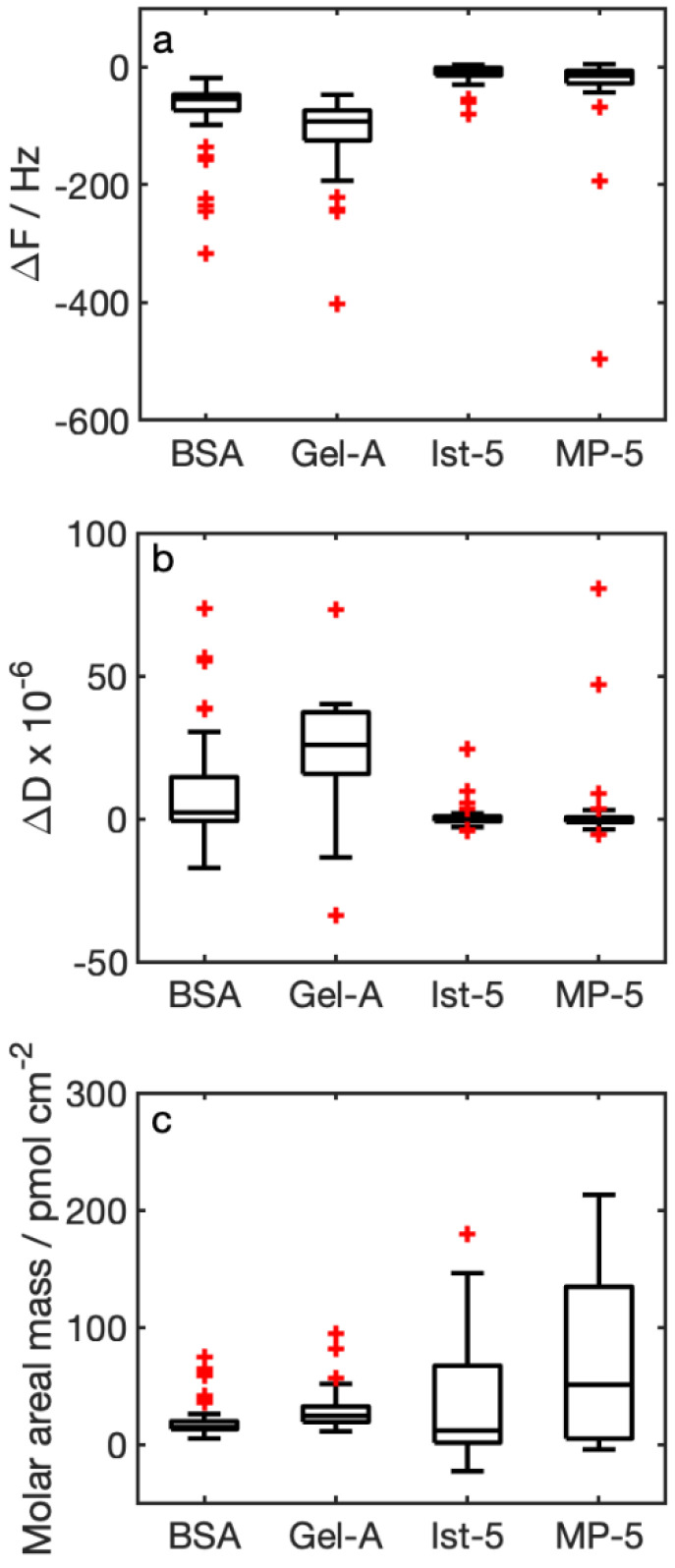
Characterization of SAM: (**a**) Δ*f* (F3), (**b**) Δ*D* (D3), and (**c**) molar areal mass calculated with the Sauerbrey model; the number of experiments for the statistical analysis is 39 for BSA, 32 for Gel-A, and 30 for Ist-5 and MP-5. Red crosses are values out of the interquartile range multiplied by 1.5.

We consider the functionalization with protein as the whole layer composed of 12-MCA + protein. QCM-D generated a plot with changes in frequency and dissipation of the experiment sequence that can be followed in real time (Figure 2). Absolute values of Δ*f* were higher in functionalization with proteins than in those with peptides (Figure 3a). At the same time, Δ*D* (Figure 3b) was higher for proteins than peptides. While for peptides dissipation there were very low values (~0), for Gel-A (3.2 × 10^−5^) and BSA (1.5 × 10^−5^) it reached values slightly higher than values recommended for the application of the Sauerbrey model. This indicates that the peptide adlayer has a rigid behavior, while proteins tend more towards a viscoelastic behavior. Median values of the molar areal mass calculated with the Sauerbrey equation (Figure 3c) were quite similar in all cases (median values are BSA 15 pmol cm^−2^, Gel-A 24 pmol cm^−2^, Ist-5 12 pmol cm^−2^, MP-5 51 pmol cm^−2^).

### 3.2. Watery Samples Detection

**Figure 4:**
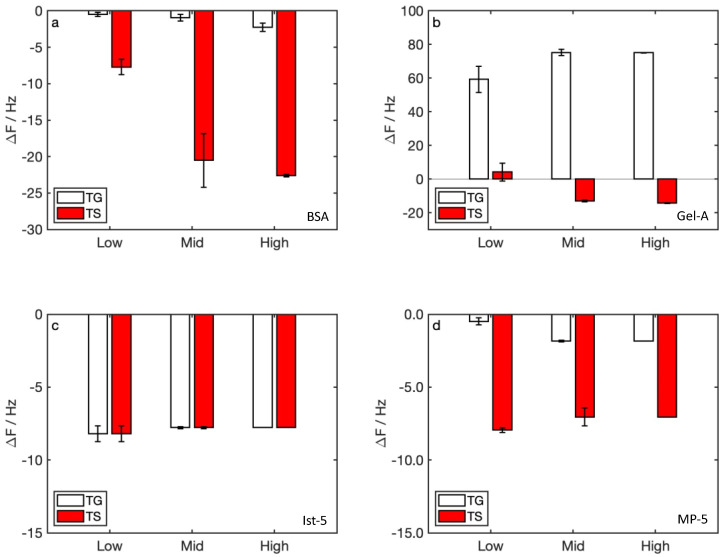
Detection of polyphenols in watery solutions: Δ*f* vs. sample concentration (see Table 1) in (**a**) BSA, (**b**) Gel-A, (**c**) Ist-5, (**d**) MP-5.

Δ*f* measured for watery samples (Figure 4) indicated, in most cases, negative values due to mass loading, and some positive data in the detection of TG with Gel-A (Figure 4b). We registered significant Δ*f* after mid-concentration sample injection only in protein-functionalized sensors, and not in peptide-functionalized sensors.

**Figure 5:**
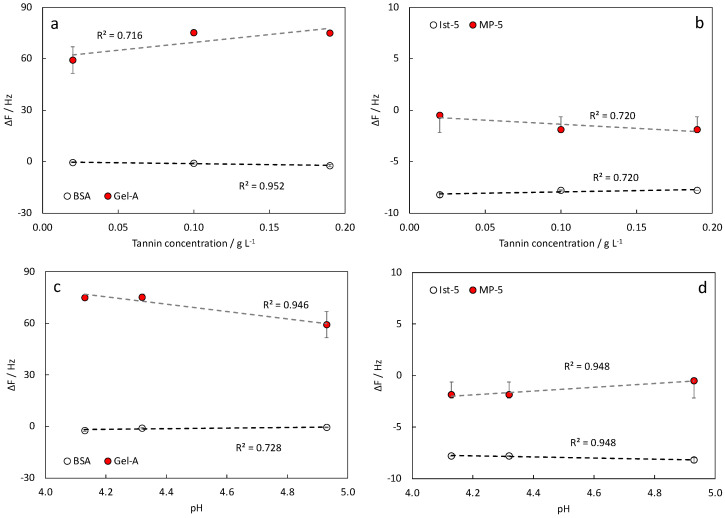
Detection of polyphenols in watery solutions containing TG: Δ*f* vs. tannin concentration in (**a**) proteins, (**b**) peptides; Δ*f* vs. pH in (**c**) proteins, (**d**) peptides.

Focusing on TG-based samples, the Δ*f* plotted against tannin concentration (Figure 5a,b) and sample pH (Figure 5c,d), showed that data might follow a linear trend in all cases. The same behavior was not verified for TS-based samples (data not shown).

### 3.3. Winery Samples Detection

**Figure 6:**
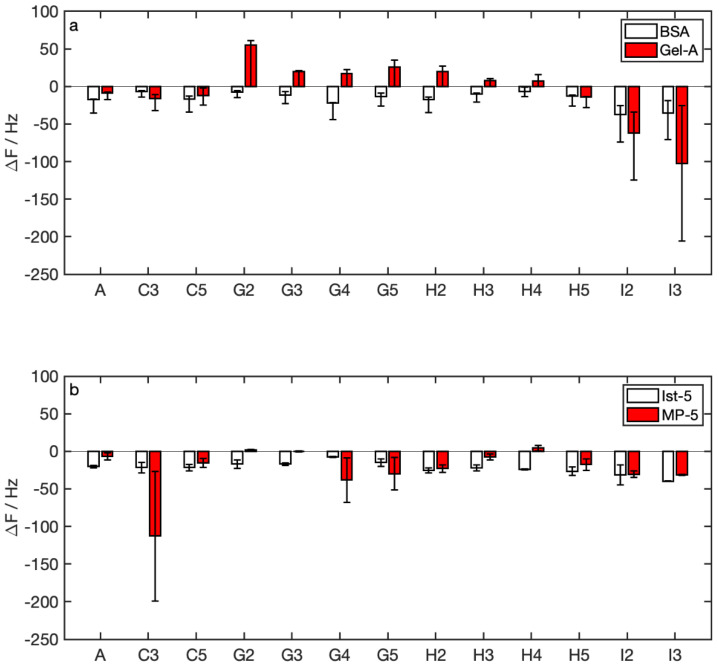
Detection of polyphenols in winery solutions: Δ*f* vs. sample nomenclature in (**a**) proteins, (**b**) peptides.

Δ*f* measured for winery samples (Figure 6) was quite in line with those measured in watery samples. We measured positive Δ*f* for samples with added TG (sample families G and H, see Table 2) with Gel-A, while the same samples provided negative Δ*f* with BSA (Figure 6a). Δ*f* measured with peptides was negative in all cases (Figure 6b).

**Figure 7:**
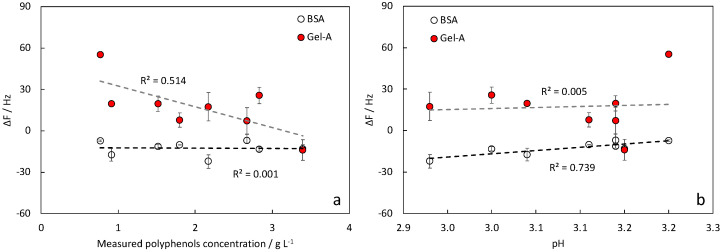
Detection of polyphenols in samples with gallic tannins added (samples G and H, Table 2): (**a**) as a function of measured polyphenols concentration, (**b**) as a function of measured pH.

Δ*f* values measured for samples G and H were aggregated and plotted against the measured polyphenol concentration (Folin–Ciocalteu method) and pH (Figure 7). We found high correlations between values obtained with Gel-A vs. polyphenol concentration and between values obtained with BSA and pH.

## 4. Discussion

Absolute values of Δ*f* measured for functionalizing adlayers were higher for proteins than for peptides (Figure 3). This could be related to the different molecular weights of molecules used for the functionalization. However, we calculated similar values for molar areal masses, indicating that the functionalization procedure is sufficient to saturate the whole crystal surface and thus leads to comparable results. This makes data obtained in sample analysis comparable to each other.

Δ*f* measured for watery samples with BSA were, in all cases, negative (Figure 4a). This represents a common case in QCM-D analysis, in which negative Δ*f* are related to the increase of deposited mass due to the interaction between sample and functionalization. With this protein, we measured increasing absolute values of Δ*f* in all cases. This indicates that the functionalization adlayer does not reach a saturation with the amounts of tannins loaded. The same effect was not verified with TS, for which the absolute values of Δ*f* in the second and in the third measures were quite similar. The different saturation limits detected for TG and TS could be due to sample concentrations, which were higher for TS that TG. Thus, the saturation of the BSA functionalization adlayer was not directly comparable in this case. In measures obtained with Gel-A-functionalized sensors, we found positive Δ*f* for TG, and a saturation for the more concentrated sample (Figure 4b). The positive Δ*f* can be explained on the basis of the interactions occurring between Gel-A and gallic tannins. As previously stated, proteins can form hydrogen bonds between prolines and functional groups of tannins. Such bonds form an interpenetrating network (IPN), in which macromolecules are physically crosslinked by interactions promoted by tannins. The formation of an IPN makes the adlayer more rigid, and part of the hydration water can be expelled, causing the increase of frequency oscillation in the sensor. The literature already clarifies the role of proline units in the interactions between food proteins and tannins [58]. This intermolecular interaction leads to the formation of a physical crosslinking of proteins [59] and, consequently, to the formation of insoluble protein–tannin complexes [60]. The blocking of hydrophilic amino acid residues (in proteins tested in this work, prolines) with the interacting molecules (tannins) is involved in the protein solubilization [61] and such kind of protein solubility modification causes the displacement of water molecules after the arising of hydrophobic interactions (hydrophobic effect) [62].

In the case of TS samples, which contained higher amounts of tannins relative to TG, the positive Δ*f* occurred only for the sample with the lower concentration, then we hypothesized a mass loading, and negative Δ*f*, for more concentrated samples. In this case, the effect of dehydration was covered by the mass loading. According to this hypothesis, BSA does not form an IPN with tannins, although the interaction would be similar to that formed in the Gel-A functionalization. The different behavior of selected proteins can be explained on their different isoelectric point (BSA 4.7 [63], Gel-A 9.0 [64]). In the range of sample pH, BSA works close to its isoelectric point, and then it has a neutral charge favoring the hydrophobic interactions, while Gel-A is under its isoelectric point and becomes positively charged. The different macromolecular charge implies a different interaction with tannins, and the formation of an IPN in BSA is hampered. In measures with peptide-functionalized sensors, we found negative Δ*f*, both for Ist-5 and MP-5, for TG and TS (Figure 4c,d). While in Ist-5 sensors the saturation seemed to be reached after the first sample injection, in MP-5 we had increasing signals for TG measures. As a general rule, we find saturation concentrations higher for proteins than for peptides.

The detection of polyphenols in winery samples shows some important similarities with the analysis of watery samples in measures with protein-functionalized sensors (Figure 6). Winery samples belonging to families G and H were added with the same tannins contained in the sample indicated as TG. Δ*f* obtained with samples G and H resulted negative for BSA and positive for Gel-A, in compliance to watery sample analysis. Values of Δ*f* are not directly comparable between watery and winery samples because of the different tannin concentrations.

Focusing our analysis only on G and H samples, Δ*f* obtained with sensors functionalized with Gel-A could be correlated with the overall polyphenol concentration and not with the pH, while those obtained with BSA-functionalized sensors correlated with the pH and not with the concentration (Figure 7). This suggests that Gel-A-functionalized sensors are not affected by the pH, making the sensor more reliable. In contrast, BSA-functionalized sensors were affected by the pH under the isoelectric point. However, due to the nonlinear correlation between pH and tannin concentrations in winery samples, the functionalization with BSA seems to be unsuitable for such measures. Appreciable Δ*f* was obtained with samples I with protein-functionalized sensors, indicating that both functionalizations can be used for proanthocyanidins detection. Gel-A, in particular, was able to discriminate between gallic tannins and proanthocyanidins on the basis of the positive or negative Δ*f* obtained.

Sensors functionalized with peptides gave appreciable Δ*f* in measures with winery solutions, indicating that interactions between tannins and probe molecules were established. However, the complex wine matrix, also containing ethanol, sugar, and wine proteins, makes the detection more difficult. However, the potential interaction of the whole components of wine on our developed functionalizations is not negligible. To date, we know that the ethanolic content has an important effect only on measure transient, so this compound should be not considered the most responsible for a potential interference. On the other hand, the literature reports that proteins and sugars can strongly interact [65]. A deeper characterization of developed functionalizations involves the analysis of BSA and Gel-A behaviors in the presence of wine sugars (mainly, glucose and fructose, and sucrose to a minor extent). Moreover, proteins are generally accounted for the nonspecific signal in gravimetric sensors, thus suitable strategies to reduce nonspecific adsorption over the free Au surface of the sensitive element (e.g., by increasing hydrophilicity) and the functionalization adlayer (e.g., by blocking with specific molecules) are also needed.

Conventional analytical methods (e.g., HPLC) are well consolidated techniques for tannin concentration quantification [66]. Moreover, results from conventional analytical methods are considered more reliable than those obtained through other techniques, but they also require an elaborated preparation and long analysis time and are often unsuitable for real time analysis [67]. Whilst acoustic sensors show a lower sensitivity in respect to consolidated analytical methods, they can give some easy-to-understand quantitative information. As an example, the tannins/proanthocyanidins balance is an indicator of the astringency, which is a very important property in the characterization of wines. Our experiments showed that this technology can be used as a fast indicator of the potential mouthfeel generated during vinification.

## 5. Conclusions

Selected proteins and peptides used in this work for the functionalization of QCM crystals show a potential in tannins analysis. Surprisingly, protein-based functionalizations are not affected by the wine matrix, thus measures in winery samples are analogous to those obtained for watery samples. Functionalizations with proteins work better than those with peptides, although selected peptides show interesting potential in discriminating proanthocyanidins versus gallic tannins, in particular MP-5.

Taken together, our results indicate that different families of tannins and concentrations can be effectively detected by the proposed functionalization strategies in a sensing platform (the QCM-D) that can be easily miniaturized for portable analysis. Outcomes are promising and pave the way for the exploitation of such devices for precision oenology.

## Figures and Tables

**Table 1:** Aqueous solutions used for QCM-D measures: sample nomenclature, commercial tannin blend dissolved in water, polyphenol concentration (calculated), and measured pH.

Sample Nomenclature	Commercial Tannin Blend Added	Polyphenol Concentration/g L^−1^	pH
TG-Low	TANIN GALALCOOL	0.02	4.93
TG-Mid		0.10	4.32
TG-High		0.19	4.13
TS-Low	TANIN VR SUPRA	0.07	5.76
TS-Mid		0.26	4.91
TS-High		0.52	4.91

**Table 2:** Winery solutions used for QCM-D measurements: sample nomenclature, commercial tannin blend added to the base wine, polyphenol concentration measured via UV spectrophotometry (reading at 280 nm) and with the Folin–Ciocalteu method, proanthocyanidins concentration measured via the Bate–Smith method, measured pH; each value represents mean ± standard deviation (*n* = 3).

Sample Nomenclature	Commercial Tannin Blend Added	Polyphenol Concentration (g L^−1^ Gallic Acid Equivalent)		Proanthocyanidins Concentration /mg L^−1^ Epicatechin Equivalent	
		UV/g L^−1^	Folin–Ciocalteu		pH (±0.05)
A	-	0.14 ± 0.01	0.22 ± 0.01	50 ± 2	3.16
C3	TB	2.64 ± 0.02	2.21 ± 0.09	45 ± 2	3.34
C5		5.47 ± 0.14	4.14 ± 0.07	55 ± 2	3.15
G2	TG	0.96 ± 0.01	0.77 ± 0.01	42 ± 1	3.20
G3		2.06 ± 0.08	1.52 ± 0.07	39 ± 1	3.14
G4		3.12 ± 0.12	2.17 ± 0.10	38 ± 3	2.93
G5		4.26 ± 0.10	2.83 ± 0.01	64 ± 3	3.00
H2	TGSP	1.03 ± 0.03	0.91 ± 0.01	24 ± 4	3.04
H3		2.13 ± 0.09	1.80 ± 0.09	36 ± 2	3.11
H4		3.33 ± 0.04	2.67 ± 0.07	45 ± 6	3.14
H5		4.34 ± 0.10	3.39 ± 0.04	53 ± 5	3.15
I2	TGR	0.93 ± 0.03	1.74 ± 0.03	824 ± 1	2.93
I3		2.01 ± 0.01	3.81 ± 0.11	1702 ± 6	2.89

## Data Availability

The data presented in this study are available on request from the corresponding author.
